# A modified SEIR model to predict the behavior of the early stage in coronavirus and coronavirus-like outbreaks

**DOI:** 10.1038/s41598-021-95785-y

**Published:** 2021-08-11

**Authors:** Wilfredo Angulo, José M. Ramírez, Dany De Cecchis, Juan Primera, Henry Pacheco, Eduardo Rodríguez-Román

**Affiliations:** 1 Escuela Superior Politécnica del Litoral, ESPOL, Facultad de Ciencias Naturales y Matemáticas, Campus Gustavo Galindo Km. 30.5 Vía Perimetral, P.O. Box 09-01-5863, Guayaquil, Ecuador; 2 School of Physical Sciences and Nanotechnology, Yachay Tech University, 100119 Urcuqui, Ecuador; 3 Facultad de Ingeniería Agrícola, Departamento de Ciencias Agrícolas, Universidad Técnica de Manabí, Sede Lodana, Manabí Ecuador; 4 Departamento de Física, Universidad del Zulia, Facultad Experimental de Ciencias, Maracaibo, Venezuela; 5 Center for Microbiology and Cell Biology Instituto Venezolano de Investigaciones Científicas, Caracas, 1020A Venezuela

**Keywords:** Public health, Epidemiology

## Abstract

COVID-19 is a highly infectious disease that emerged in China at the end of 2019. The COVID-19 pandemic is the first known pandemic caused by a coronavirus, namely, the new and emerging SARS-CoV-2 coronavirus. In the present work, we present simulations of the initial outbreak of this new coronavirus using a modified transmission rate SEIR model that takes into account the impact of government actions and the perception of risk by individuals in reaction to the proportion of fatal cases. The parameters related to these effects were fitted to the number of infected cases in the 33 provinces of China. The data for Hubei Province, the probable site of origin of the current pandemic, were considered as a particular case for the simulation and showed that the theoretical model reproduces the behavior of the data, thus indicating the importance of combining government actions and individual risk perceptions when the proportion of fatal cases is greater than $4\%$. The results show that the adjusted model reproduces the behavior of the data quite well for some provinces, suggesting that the spread of the disease differs when different actions are evaluated. The proposed model could help to predict outbreaks of viruses with a biological and molecular structure similar to that of SARS-CoV-2.

## Introduction

COVID-19 is a highly contagious respiratory disease caused by a new virus named SARS-CoV-2. The first COVID-19 contagion was reported in Wuhan, China, in early December 2019. During the following weeks, the disease spread rapidly in mainland China and other countries, leading the World Health Organization (WHO) to declare COVID-19 a pandemic on March 11, 2020^1^. The pandemic declaration was supported by a large number of cases and deaths. Based on data from Johns Hopkins University, more than 21.2 million cases worldwide and 767 thousand global deaths were reported by late May 2020 the date when the authors started this work. Since then, these values have risen to more than 170 million cases and 3.5 million deaths^2^.

To reduce the spread of the virus, the countries affected by the pandemic adopted sanitary and social distancing measures, reduced traffic, and banned any activity that involved a concentration of people, especially in confined spaces. Previous studies that correlated health data with past flu pandemics show that school lockdowns and the human response caused by the associated risks may explain the reduction in the disease propagation rate^3^. Similar studies using data on other coronaviruses were performed by Kissler et al.^4^. Tian et al.^5^ present research based on correlated health data during the first fifty days of the COVID-19 epidemic in China, and they report that the measures adopted by citizens impacted the control of disease spread. The most effective measures examined in Tian H. et al.^5^ and the measures that provided plausible explanations for the reduced propagation rates are the suspension of city public transportation, closure of entertainment venues, and banning of public reunions. Although the conclusions from this study in China indicate that the emergency response delayed and reduced the COVID-19 epidemic, the analysis does not reveal a clear cause-effect relationship between the impact of actions taken by citizens and the rate of disease propagation. A method of establishing the causes consists of combining statistical analysis and mechanistic mathematical models^6^. He et al.^7^ propose a mechanistic mathematical model that incorporates the effect of school closures, human behavior responses, and weather changes as the most plausible actions from a health data correlation study about the flu pandemic of 1918 in London^3^. Lin et al.^8^ use a modified version of the method introduced by He et al.^7^ to simulate the spread of COVID-19 in the city of Wuhan, China, thus emphasizing the effects of the measures adopted by the government and the individual reaction on the risk associated with the disease infection rate. They use this model because of the similarities between both diseases regarding the spreading velocity. However, they eliminate the effect of weather changes because there is no evidence about its relationship with the COVID-19 infection rate. The benefits of the proposed model are related to its consideration of zoonotic transmission and high migration of people during a short period as well as government measures and individual actions. The results of the simulations show a disease spread tendency consistent with the data on infected individuals reported in the city of Wuhan from January 27 to mid-February 2020. Nevertheless, the authors do not report simulations with extended periods that would allow us to verify the mechanistic model validity regarding the most recent data and the correlation study published recently by Tian et al.^5^.

In the present work, we intend to model the early stage of the spread of COVID-19 (we started this research in February 2020 and finished the modeling and writing in May 2020) using a modified version of the simple SEIR (susceptible-exposed-infected-recovered^9–11^) and coupled it with the mechanistic model on disease infection rates proposed by He et al.^7^. For this infection rate, we eliminated the effect of weather changes but incorporated the effects studied by Tian et al.^5^ as a function of time and individual reactions to deaths, which is one of the model parameters. Our purpose is to show predictions about the disease spread in the short and initial epidemic phases for the entire province of Hubei, China, and compared these finding with the health data reported for their respective periods. Although the complete model is not sophisticated, the results obtained in our simulations show an acceptable and valuable match with the health data reported for Hubei for the initial 90-day period. Hence, we consider that the results can be used by decision-makers to plot and implement policies, as well as contingency plans, to face possible new epidemic outbreaks in the early stage. In the near future, we plan to add new features to discuss more realistic scenarios, not only for COVID-19 but also for other diseases caused by other viruses that may occur in the future.

At the time of writing this paper, different variants have arisen during the pandemic. These variants might differ substantially in the characteristics that affect the dynamics of the simulations, for example, the transmission rate or the case fatality proportion^12^. In addition, the values of the adjusted parameters are appropriate for the data provided in a particular case, and the model describes the mean behavior of the state variables. Thus, the results given for a model simulation represent the behavior of specific data provided at a specific time. Hence, we consider the procedure to be applicable to other data sets as a framework, and comparisons of these data sets with the obtained data provides a measure of the method’s effectiveness. The authors suggest that the methodology should be applied in future works for other regions.

This paper is structured as follows. In “Mathematical model”, we introduce the mathematical aspects of the model, defining all state variables and equations. In “Data used in the model”, we describe the health data used to fit the parameters. In “Numerical simulations”, we describe all the simulation runs. In “Results and discussion”, we provide a discussion. Finally, in “Conclusions”, we present the conclusions of this work.

## Mathematical model

We adopt the classical SEIR (**S**usceptible-**E**xposed-**I**nfected-**R**ecovered) framework as a baseline. In the SEIR model, the total population, which is represented by the variable *N*(*t*) for all $t\ge 0$, is regrouped into sets of individuals who are seen as units^11^. In this sense, the variable *S*(*t*) represents the number of persons susceptible to infection, *E*(*t*) represents the number of persons exposed to the infection, *I*(*t*) represents the number of persons infected after exposure, and *R*(*t*) represents the number of persons recovered after infection. Additionally, we add the variable *D*(*t*), which represents those patients who do not recover from infection, are not infectious but ultimately die, and the variable *M*(*t*), which represents the number of deaths from the disease. All these variables, of course, depend on time.

We consider the parameter $\phi$ related to the case fatality proportion (CFP) leaving the infected unit, which represents the individuals who are not susceptible to becoming infected again because they are in the process of dying within time $g ^ {-1}$ and whose time until death is measured by the rate of change of the variable *D*(*t*). This fraction of individuals is dynamically accounted for by the rate of variation with respect to time of the variable *M*(*t*).

We now describe the equations governing the system in terms of the normalized variables *s*(*t*), *e*(*t*), *i*(*t*), *r*(*t*), *d*(*t*) and *m*(*t*). In other words, we rescale the variables described above with respect to the total population, which allows the suitable management of the model from a numerical perspective without the effects of differences in scale. In this sense, we assume that the change in the *s*(*t*) fraction decreases proportionally to that of $[\beta _0 s(t) f(t) + \beta (t) s(t) i(t)]$, where $\beta (t)$ is the transmission rate, which converts *s*(*t*) into *e*(*t*), and before spending a mean amount of time, it converts to $\sigma ^{-1}$ into the unit of infected individuals *i*(*t*). The zoonotic transmission is implemented with a stepwise function *f*(*t*). Denoting the derived operator with respect to time by $\partial _{t}$, our model is described as follows:

1 $$\begin{aligned} \partial _t\, s(t) + \beta _0\, s(t) f(t) + \beta (t)\, s(t) i(t) + \mu \, s(t)=0, \end{aligned}$$

2 $$\begin{aligned} \partial _t\, e(t) - \beta _0\, s(t) f(t) - \beta (t)\, s(t) i(t) + (\mu + \sigma )\,e(t)=0, \end{aligned}$$

3 $$\begin{aligned} \partial _t\, i(t) - \sigma \, e(t) + (\gamma +\mu )\, i(t)=0. \end{aligned}$$

The next layer in our model represents individuals that recovered with a rate of $(1-\phi )\gamma$. After a period of illness $\gamma ^{-1}$ (mean infectious period), the *i*(*t*) is converted into *r*(*t*):

4 $$\begin{aligned} \partial _t\, r(t) - (1-\phi )\, \gamma \, i(t) + \mu \, r(t)=0. \end{aligned}$$

The rates of change for the variables *d*(*t*) and *m*(*t*) are given as follows:

5 $$\begin{aligned} \partial _t\, d(t) + g\, d(t) - \phi \, \gamma \, i(t)=0, \end{aligned}$$

6 $$\begin{aligned} \partial _{t}\, m(t) - g\, d(t)= 0. \end{aligned}$$

This model is complemented by the following equation used to determine the total population at each instant of time $t>0$:

7 $$\begin{aligned} \partial _t N(t) + \mu (t)\,N(t)[1 - d(t) - m(t)]= 0. \end{aligned}$$

The total individual population *N*(*t*) decreases for a positive migration rate $\mu (t)$ and increases for a negative migration rate (the population model remains constant with $\mu =0$). Although we consider the parameter $\mu$ as a time-dependent stepwise function, as shown in Table 1, we remove the time dependency for simplicity of notation.

**Table 1:** Parameters of the Wuhan COVID-19 outbreak models.

Quantity	Description	Type	Value	Initial value	References
*F*(*t*)	Zoonotic/day	Stepwise function	{0, 10}	10	–
*N*(*t*)	City population	Computed	–	14 MM	South China Morning Post (2020)^13^
*s*(*t*)	Susceptible	Computed	–	0.9999	–
*e*(*t*)	Exposed	Computed	–	0.0001	–
*i*(*t*)	Infected	Computed	–	0	–
*r*(*t*)	Recovered	Computed	–	0	–
*d*(*t*)	Expected to die	Computed	–	0	–
*p*(*t*)	Perception of risk	Computed	–	0	–
$\kappa$	Strength of response	Constant	(0, 1117.3)	–	He et al. (2013)^3^
$\alpha$	Governmental action strength	Stepwise function	{0, 0.4239, 0.8478}$^a$	0	Lin et al. (2020)^8^
$\beta _0$	Baseline transmission rate	Stepwise function	{0.5944, 1.68}	0	Lin et al. (2020)^8^
$\mu$	Emigration rate	Stepwise function	{0, 0.0205, 0}	0	South China Morning Post (2020)^13^
$\sigma ^{-1}$	Mean latent period	Constant	3 days	0	Wu et al. (2020)^14^
$\gamma ^{-1}$	Mean infectious time	Constant	5 days	0	Wu et al. (2020)^14^
$\lambda ^{-1}$	Mean time of public reaction	Constant	11.2 days	0	He et al. (2013)^3^ & Lin et al. (2020)^8^
$g^{-1}$	Mean time in unit *d*	Constant	8	0	He et al. (2013)^3^
$\phi$	Case fatality proportion	Constant	(0.5%, 20%)	0	–

^a^From January 23 to January 29, 2020, $\alpha =0.4239$. After January 30, 2020, $\alpha =0.8478$.

All dynamical quantities in our novel SEIR model, which is normalized by *N*(*t*), satisfy the following relationship:

8 $$\begin{aligned} s(t)+e(t)+i(t)+r(t)+d(t)+m(t) = 1 - \mu (t)\,\left[ 1 - d(t) - m(t) \right] . \end{aligned}$$

**Figure 1:**
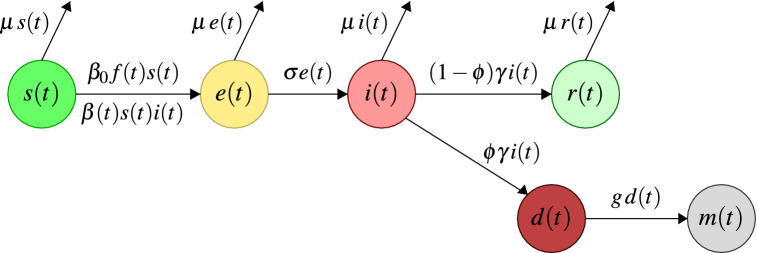
Diagram of the modified SEIR model.

Figure 1 shows the flow diagram that summarizes the model.

We use the transmission rate function $\beta (t)$ defined by He et al.^3^ that incorporates the impact of governmental actions (all actions that impact mobility) and the decreasing contacts among individuals in response to the proportion of deaths or severe cases (i.e., the severity of the epidemic) as follows:

9 $$\begin{aligned} \beta (t):=\beta _0(1-\alpha )\left[ 1- p(t)\right] ^{\kappa }, \end{aligned}$$

where the quantity $(1-\alpha )$ represents the seasonality of governmental actions (quarantine, airport closure, shopping center closure, social distancing, curfews, etc.)^8^ for all $\alpha \in [0,1]$, and $\kappa$ is a parameter that represents the intensity of perception of the risk *p*(*t*) that the individuals exhibit during the pandemic. This public perception of risk is modeled as follows:

10 $$\begin{aligned} \partial _{t} p(t) + \lambda p(t) - g d(t)= 0, \end{aligned}$$

where $\lambda ^{-1}$ is the mean duration of impact of deaths on public perception. For instance, the spread of a disease without any action from part of the susceptible population is $\kappa =0$ (naive spread). Governmental actions, such as quarantine and lockouts, are considered when $\alpha \ne 0$. A goal of this model is to analyze the effects of individual reactions, public risk perceptions, and governmental action on the dynamics of susceptible populations to suffer the spread of a disease, in this case, COVID-19.

## Data used in the model

**Figure 2:**
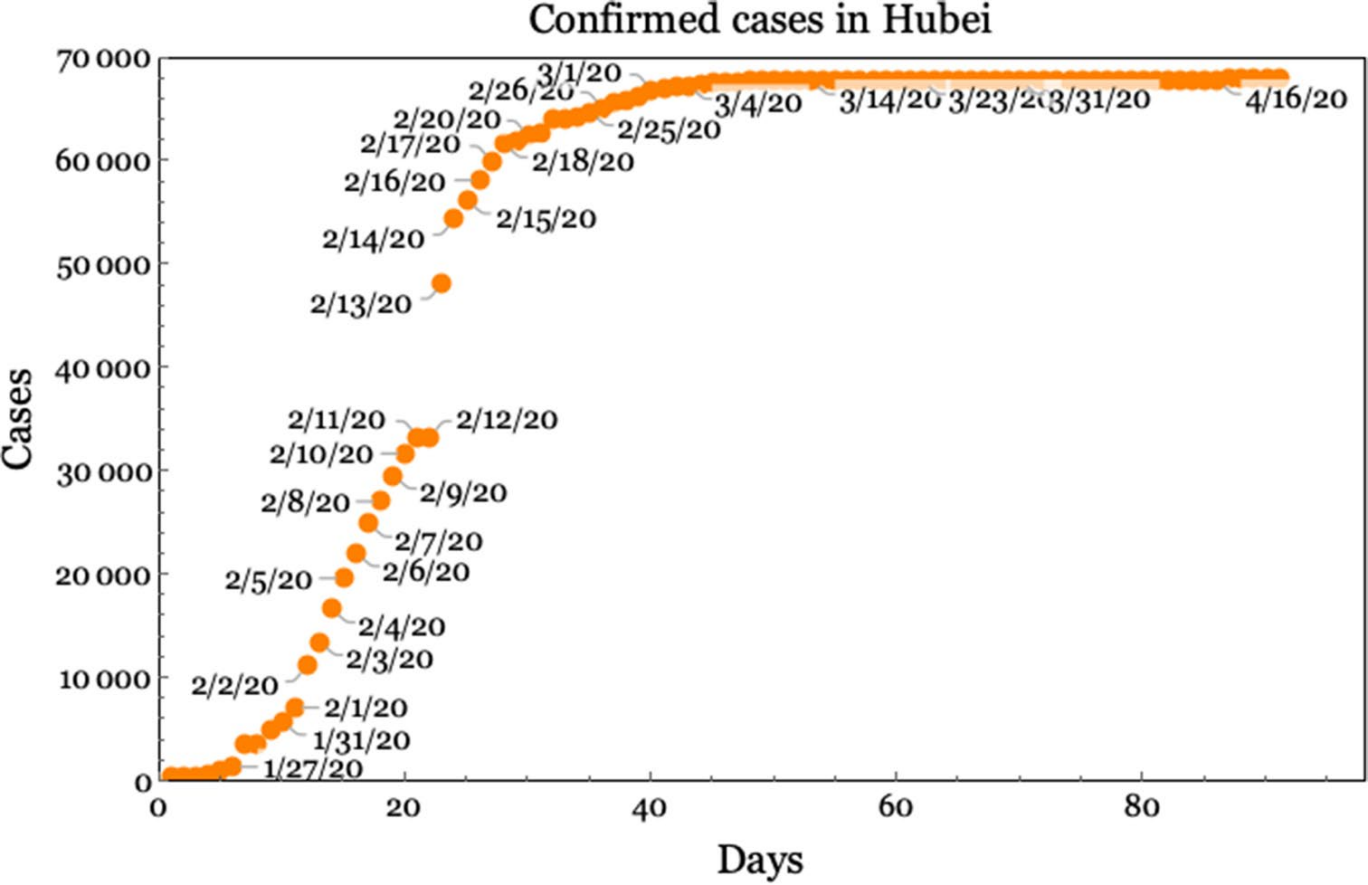
Reported cumulative daily confirmed COVID-19 cases in the province of Hubei since January 22, 2020. Source: CSSE, Johns Hopkins University.

We use the data provided by the COVID-19 Data Repository by the Center for Systems Science and Engineering (CSSE) at Johns Hopkins University^2^. From this repository, we use the time series of confirmed cases, number of deaths and number of recovered individuals in China’s 33 administrative dependencies from January 22, 2020, until 90 days later. For example, Fig. 2 depicts a plot of cumulative cases in the province of Hubei.

**Figure 3:**
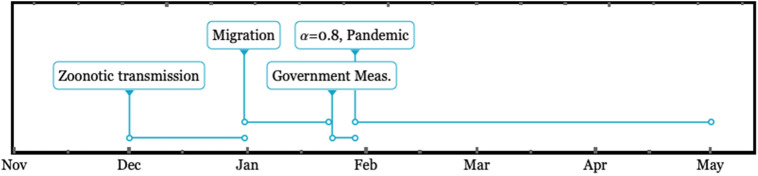
Timeline of the spread of COVID-19 and governmental actions taken in Wuhan (China) surrounding the days of the outbreak in 2019. Zoonotic transmission began on December 1, 2019. Migration started on December 31, 2019, and ended on 22 January 2020. *Soft government actions* taken on 23 January and *stronger actions* taken on 29 January 2020 decreased the baseline human-to-human transmission rate $\beta _0$.

From the beginning of the outbreak, we assume the timeline given in Fig. 3. The outbreak started on December 1, 2019, in a seafood market in the city of Wuhan in Hubei^8^. Then, because of the Chinese New Year holiday, a strong migration occurred from December 31 until January 22, 2020, when the Chinese government started the first *soft control measures*, with additional *stronger governmental measures* taken on January 29 that imposed circulation control, social distancing, educational lockout, etc. In our model, following the settings by Lin et al.^8^ for the transmission function in Eq. (9), we set the parameter $\alpha$ to values of $\alpha = 0$ from December 1 until January 23, $\alpha = 0.4239$ from January 23 to January 29, and $\alpha = 0.8478$ after January 29 . The zoonotic transmission function *f*(*t*) in Eq. (1) is the normalized value of the zoonotic transmission rate *F*(*t*) defined in Table 1. For the province of Hubei, *F*(*t*) is set as a stepwise function, with the value of $F_0=10$ from December 1 until December 31 and zero the rest of the time. For the rest of the administrative dependencies, the zoonotic transmission rate is always set to zero.

### Data fitting

We fit the 33 administrative dependencies in China reflected in the aforementioned database, including the province of Hubei, which is the case study considered in this work. The data set used in our analysis consists of the first 90 days from January 22, 2020. We use the maximum-likelihood method, which closely follows the procedure used by He et al.^3^, to fit the free parameters $N_0$, $\sigma$, and $\beta _0$. The latter is split into two different base transmission rates, $\beta _{01}$ and $\beta _{02}$. The splitting of $\beta _0$ corresponds to the finding that the disease transmission rate changes in response to the different governmental actions on two different dates.

## Numerical simulations

We organize the numerical simulations into two main sets. For the first set, which is presented in subsection entitled *Model simulations*, we run our model for four different values of the CFP to elucidate its effect on the evolution of the disease spread related to government actions and the public perception of risk. For the second set, which is presented in subsection entitled *Fitting using the health data from Chinese provinces*, we perform data fitting with the health data using the maximum-likelihood method.

### Model simulations

From this set of simulations, we intend to elucidate the effect of individual reactions from the perception of risk and governmental actions on the transmission dynamic. Thus, we define three different model categories: the *naive* model, in which the disease spread is modeled via SEIR using a constant transmission rate $\beta (t)=\beta _0$ ($\alpha = 0$ and $\kappa =0$); the *individual reaction* model, which considers only the public perception of risk ($\alpha =0$ and $\kappa = 1117.3$); and the *individual and government reaction* model, which considers both the government actions and the individual reaction for perception risk ($\alpha \ne 0$ and $\kappa \ne 0$). Then, we run the three categories of models for four different CFPs: $\phi =0.2$, 0.02, 0.01, and 0.005. We label these runs as extreme, high, middle, and low CFPs, respectively. The simulations start on December 1, 2019, and run over 180 days based on the timeline in Fig. 3. In all of the runs, we monitor the evolution of *N*(*t*) according to Eq. (8).

**Figure 4:**
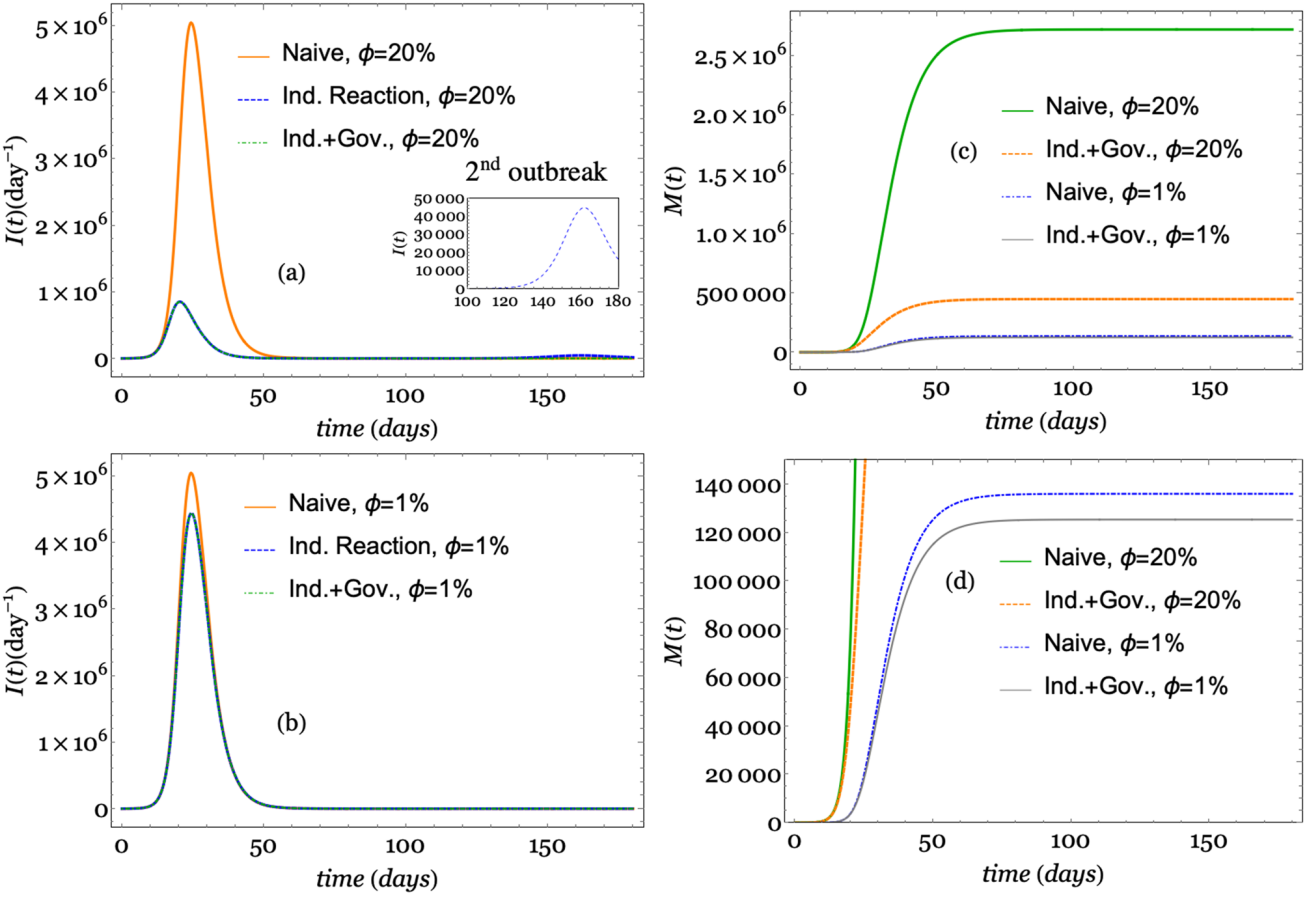
Simulations using the values of the parameters shown in Table  1 for the three different model categories. Plot (**a**) depicts the number of infections per day for the extreme CFP ($\phi =20$%). Plot (**b**) shows the number of infections per day for the middle CFP ($\phi =1$%). Plot (**c**) shows the number of deaths per day for both the extreme and middle CFPs ($\phi =20$% and $\phi =1\%$), and plot (**d**) is a magnified view of the lower lines of number of deaths.

**Figure 5:**
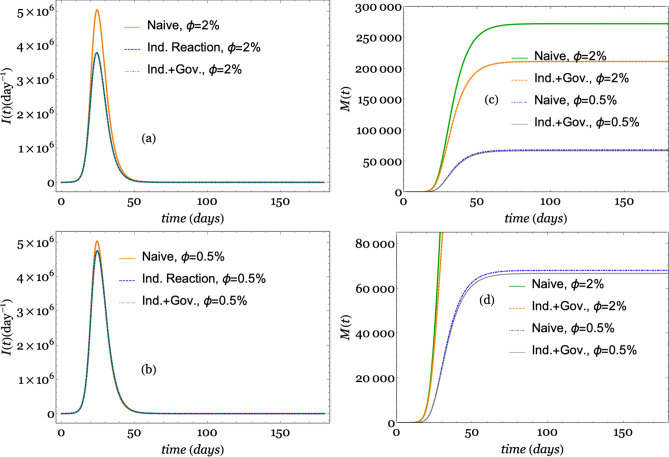
Simulations using the values of the parameters shown in Table 1 for the three different model categories. Plot (**a**) depicts the number of infections per day for a high CFP ($\phi =2$%). Plot (**b**) shows the number of infections per day for a low CFP ($\phi =0.5$%). Plot (**c**) shows the number of deaths per day for both high and low CFPs ($\phi =2$% and $\phi =0.5\%$). Plot (**d**) shows a magnification of the lower lines of number of deaths.

Figures 4 and 5 depict the results of the simulations using the values of the parameters shown in Table 1 except for those parameters established in each scenario. Figure 4 depicts the number of infections per day (*I*(*t*)) and the number of deaths (*M*(*t*)) considering the extreme and middle CFPs. In the left panel, the number of infections per day is depicted for the extreme CFP in the upper plot (a) and for the middle CFP in the lower plot (b). A second outbreak is shown in the inset of plot (a). In the right panel, the number of deaths *M*(*t*) in the naive and individual reaction together with government action models for the extreme and middle CFPs is depicted in the upper plot. A magnified view of the lower lines is shown in the lower plot (d).

Similarly, Fig. 5 depicts the results obtained by simulating high and low CFPs, and it shows the numbers of infections per day (*I*(*t*)) and deaths (*M*(*t*)). The left panel shows plots of the number of infections per day for the high CFP in the upper plot (a) and for the low CFP in the lower plot (b). The right panel shows a plot of the number of deaths (*M*(*t*)) in the upper plot (c) and a magnified view in the lower plot (d).

### Fitting using the health data from Chinese provinces

We fit the parameters shown in Table 2 using the maximum-likelihood method as described in He et al.^3^. We consider the following free parameters: the initial susceptible population, $N_0$; the inverse of the mean latent period, $\sigma$; and the baseline transmission rate, $\beta _0$. In some cases, we split this parameter into two, e.g., $\beta _{01}$ and $\beta _{02}$, to consider different dynamics for disease spread. Once the best fit of the parameters is found, we identify the $1-\sigma$ error estimation (1 standard deviation). It is worth mentioning that some of the fits are of good quality, for which we have modest to high confidence, whereas some are of low quality. Nevertheless, we present all of these fits for the sake of completeness of the survey.

**Table 2:** Parameters of the 33 provinces in China with publicly available data COVID-19 outbreak models.

Province	$N_0$	$\sigma$	$\beta _{01}$^a^	$\beta _{02}$^b^	$R^2$
$\text {Anhui}$	$7499.0\pm 1217.0$	$30.190\pm 186.9$	$0.62\pm 0.067$	–	0.91
$\text {Beijing}$	$3947.0\pm 500.8$	$60.350\pm 477.4$	$0.56\pm 0.036$	$10.75\pm 2.726$	0.78
$\text {Chongqing}$	$2330\pm 67.1$	$0.061\pm 0.003$	$28.19\pm 2.526$	–	0.97
$\text {Fujian}$	$1356 \pm 62.3$	$0.054 \pm 0.004$	$36.66 \pm 5.359$	$250000 \pm 2.8(-14)$	0.93
$\text {Gansu}$	$288.6\pm 19.1$	$2.077\pm 0.531$	$1.03\pm 0.07$	$3787\pm 3663$	0.74
$\text {Guangdong}$	$6169\pm 176$	$0.05\pm 0.003$	$31.84\pm 2.706$	−	0.97
$\text {Guangxi}$	$1164.0\pm 46.3$	$0.054\pm 0.004$	$23.61\pm 2.655$	–	0.95
$\text {Guizhou}$	$1086.0\pm 169.5$	$22.820\pm 104.8$	$0.6\pm 0.061$	–	0.91
$\text {Hainan}$	$1119\pm 200$	$20.61\pm 95.04$	$0.64\pm 0.078$	–	0.88
$\text {Hebei}$	$1999\pm 176.8$	$46.66\pm 221.6$	$0.6\pm 0.032$	$96550\pm 1.532\times 10^{-13}$	0.90
$\text {Heilongjiang}$	$4198\pm 1129$	$1.229\pm 0.832$	$0.7\pm 0.146$	$11.9\pm 6.088$	0.79
$\text {Henan}$	$8154\pm 975.6$	$43.23\pm 261.9$	$0.65\pm 0.052$	–	0.89
$\text {Hong Kong}$	$2.6\times 10^6\pm 6.9\times 10^7$	$0.0003\pm 0.006$	$221.3\pm 5691$	$3.2\pm 4.698$	0.83
$\text {Hubei}$	$1.1\times 10^6\pm$200,600	$37.26\pm 369.8$	$0.45\pm 0.04$	–	0.90
$\text {Hunan}$	$5647\pm 537.7$	$33.82\pm 113.3$	$0.68\pm 0.042$	–	0.85
$\text {Inner Mongolia}$	$905.3\pm 247.1$	$1.03\pm 0.622$	$0.69\pm 0.139$	$14.87\pm 8.254$	0.69
$\text {Jiangsu}$	$2133\pm 65.6$	$0.073\pm 0.004$	$15.74\pm 1.399$	100,000$\pm 1.239\times 10^{-14}$	0.96
$\text {Jiangxi}$	$3243\pm 99$	$0.09\pm 0.006$	$10.54\pm 0.937$	–	0.96
$\text {Jilin}$	$311.6\pm 8.8$	$0.083\pm 0.005$	$11.33\pm 0.918$	$97710\pm 1.375\times 10^9$	0.97
$\text {Liaoning}$	$839.6\pm 231.6$	$16.75\pm 96.38$	$0.67\pm 0.134$	$4.53\pm 1.841$	0.80
$\text {Macau}$	$264.8\pm 110.4$	$0.604\pm 0.479$	$0.71\pm 0.238$	$34.21\pm 46.650$	0.37
$\text {Ningxia}$	$368.4\pm 96.1$	$23.140\pm 175.3$	$0.64\pm 0.115$	–	0.80
$\text {Qinghai}$	$78.6\pm 4.9$	$35.9\pm 73.33$	$0.84\pm 0.039$	–	0.91
$\text {Shaanxi}$	$1762\pm 298.9$	$37.740\pm 291.8$	$0.64\pm 0.074$	–	0.85
$\text {Shandong}$	$9032\pm 2857$	$28.3\pm 370.7$	$0.46\pm 0.074$	–	0.74
$\text {Shanghai}$	$2567\pm 353.8$	$24.33\pm 84.53$	$0.65\pm 0.054$	$12.51\pm 3.579$	0.78
$\text {Shanxi}$	$1012\pm 157$	$14.22\pm 36.75$	$0.64\pm 0.064$	$7.34\pm 1.575$	0.83
$\text {Sichuan}$	$4656\pm 1292$	$24.94\pm 230.2$	$0.56\pm 0.096$	–	0.78
$\text {Tianjin}$	$1061\pm 99.3$	$24.32\pm 57.34$	$0.6\pm 0.03$	$10.81\pm 1.919$	0.85
$\text {Tibet}$	–	–	–	–	–
$\text {Xinjiang}$	$801.9\pm 159.9$	$25.11\pm 172.6$	$0.53\pm 0.061$	–	0.89
$\text {Yunnan}$	$1255\pm 102.7$	$32.490\pm 77.14$	$0.65\pm 0.03$	$3.49\pm 4.682$	0.80
$\text {Zhejiang}$	$7090\pm 1869$	$28.35\pm 253$	$0.7\pm 0.136$	–	0.81

^a^Human-to-human infection rate (in cases per day) when the soft governmental measures were implemented on January 23, 2020 $(\alpha =0.4239)$.

^b^Human-to-human infection rate (in cases per day) when the hard governmental measures were implemented on January 29, 2020 $(\alpha =0.8478)$.

**Figure 6:**
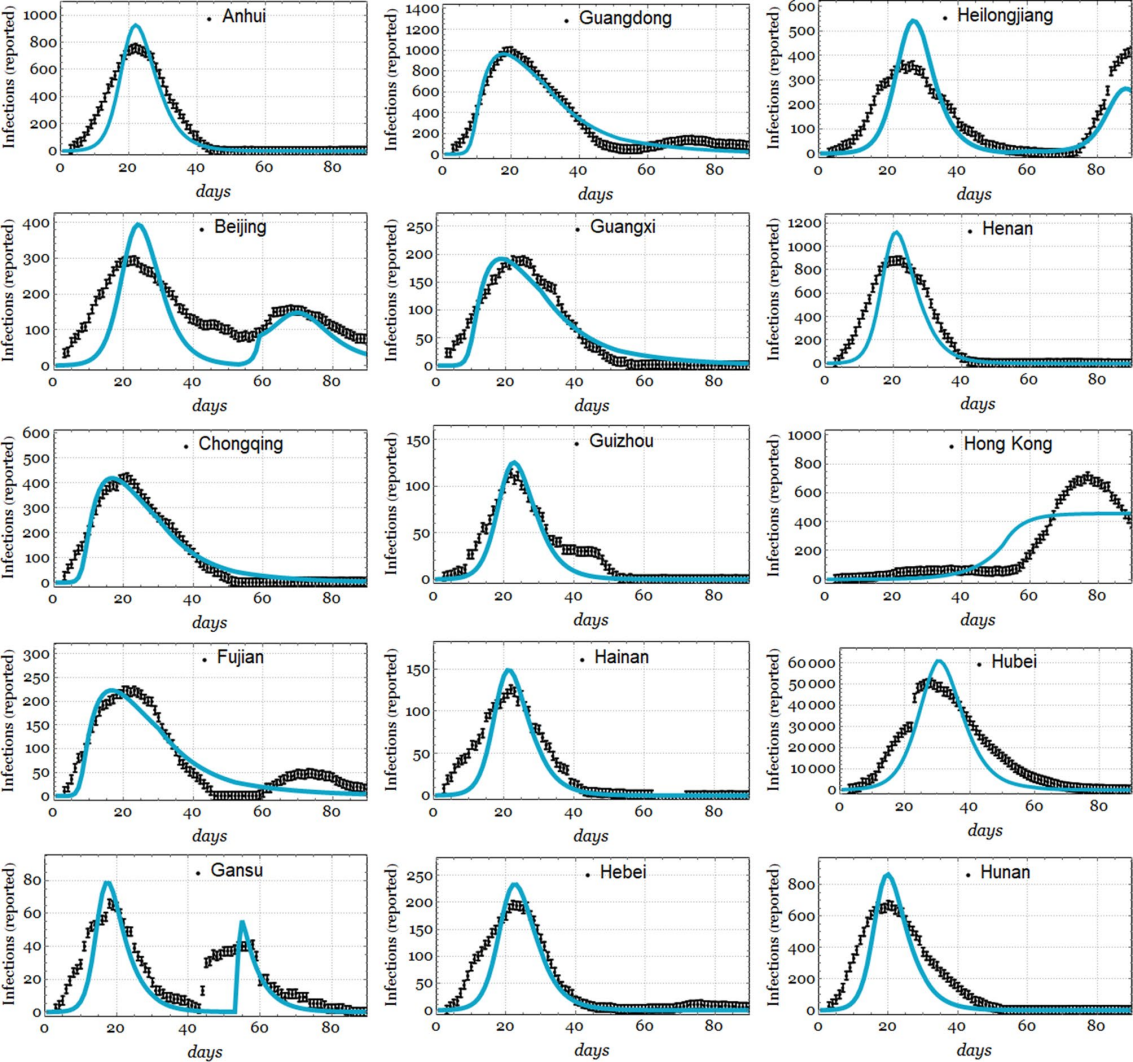
Fit of the first 15 Chinese provinces produced using the data from the CSSE repository and our model using the values of the parameters as in Table 1, with $F_0=10$ for Hubei and $F_0=0$ otherwise. Occasionally, $\beta _{02}$ has to be removed from the fit to reproduce the smaller second outbreaks seen in some provinces. We give the best fit parameters and their quality of fit ($R^2$) in Table 2.

**Figure 7:**
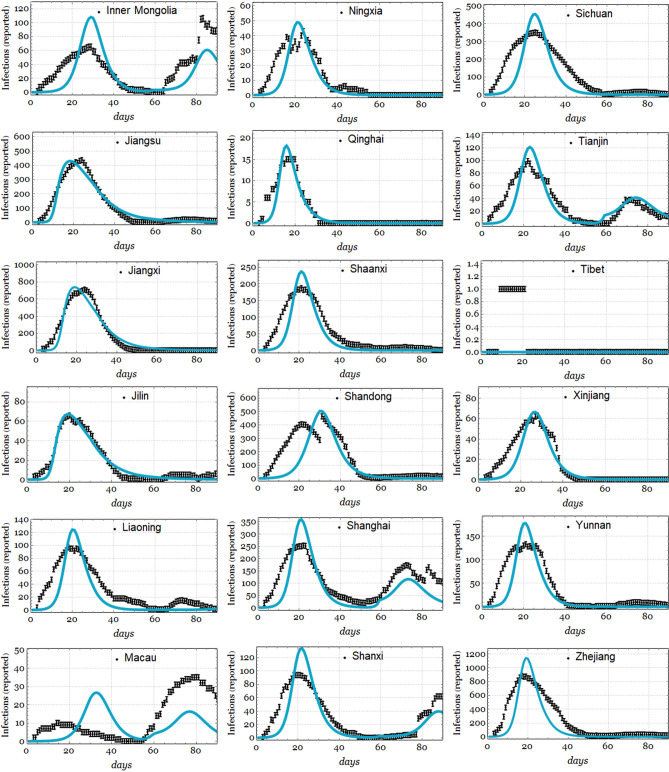
Fits from the remaining 18 to 33 Chinese provinces produced using the data from the CSSE repository and our model using the values of the parameters as in Table 1, with $F_0=10$, for Hubei and $F_0=0$, otherwise. Occasionally, $\beta _{02}$ has to be removed from the fit to reproduce the smaller second outbreaks seen in some provinces. We give the best fit parameters and their quality of fit ($R^2$) in Table 2.

**Figure 8:**
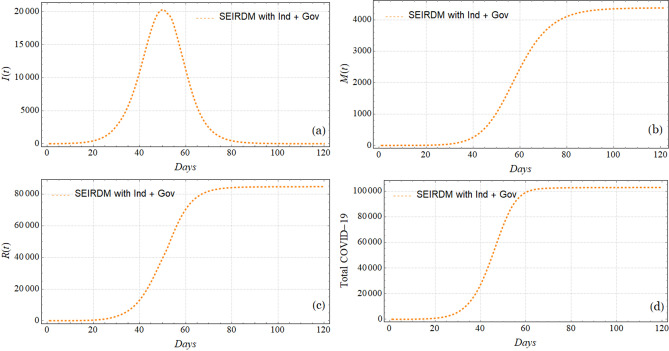
Plots of the simulation results using the data of Hubei. These simulation plots have an initial population of $N_0 = 1.14\times 10^{6}$, $\beta _{01} = \beta _{02}= 0.5$, which was previously estimated (see Table 2), and CFP $\phi =4.5\%$, which was very similar to the actual value. The rest of the parameters are the same as in Table 1, and the timeline given Fig. 3 is used. Plot (**a**) shows the number of infected individuals (*I*(*t*)), plot (**b**) shows the accumulated deaths *M*(*t*), plot (**c**) shows number of recovered individuals *R*(*t*), and plot (**d**) shows the accumulated cases.

Figure 6 shows the results of fitting the number of infections per day *I*(*t*) for a subset of 15 provinces in China, and the remaining results are shown in Fig. 7. Figure 8 depicts the results of the model using the Hubei data, using $N_0 = 1.14\times 10^{6}$, $\beta _{01} = \beta _{02}= 0.5$, which was estimated in the data fitting, and CFP $\phi =4.5\%$, which is very similar to the actual value. The rest of the parameters are the same as in Table 1. Plot (a) represents the number of infected individuals *I*(*t*), plot (b) indicates the number of deaths *M*(*t*), plot (c) indicates the number of recovered individuals *R*(*t*), and plot indicates (d) the total cumulative cases.

## Results and discussion

In Figs. 4 and 5, we observe a reduction in the number of infections per day based on a comparison of the naive model, the individual reaction model and the individual reaction with government reaction model. The findings show that individual reactions and government measures led to a reduction in the number of infections per day. The curve representing the naive simulation did not show variations with respect to the changes in the CFP parameter $\phi$, while these changes produced a remarkable effect on the other two models. The simulations showed that a larger value of parameter $\phi$ corresponds to a lower peak in the number of infections per day. The reduction with respect to the naive simulation is approximately 5:1 when $\phi =20\%$, while it is barely perceptible when $\phi =0.5$%.

At first glance, one can assume that the CFP is not related to the number of infected individuals. However, the higher the number of deaths is, the more cautious individuals become because the risk perception increases; thus, the possibility of contagion decreases. Equation (5) indicates that our model reproduces this behavior in a consistent manner. In fact, the variable *i*(*t*) is directly related to the variable *d*(*t*) (and then to *m*(*t*)) and inversely related to the parameter $\phi$. Thus, for a higher value of $\phi$, a lower value of *i*(*t*) is obtained.

In Fig. 4a, when $\phi =10\%$, we also observe another small peak produced by the simulations around day 160, although this is only observed when only the individual reaction is considered. In contrast, we do not observe this second peak for the other model categories with a lower value of $\phi$. These secondary peaks might represent rebounding outbreaks; thus, the model consistently reproduces this behavior that we expect in the short and long term, which is similar to that observed for other infectious diseases^3^. It is worth mentioning that there is almost no difference in the curves when we add the government measurements in addition to the individual response, which indicates that government action does not have a significant impact on the change in the curve. From our simulations, we understand that it is more important to consider actions taken by citizens than actions taken by the government that are not assimilated by individuals. Such a perspective centers contagion prevention mainly on individuals instead of measure enforcement.

With respect to the number of deaths (*M*(*t*)), the simulations are also consistent. Figures 4 and 5 (plots (c) and (d)) clearly show that a larger the CFP corresponds to a higher number of deaths. Additionally, we assess the influence of public risk perception and government action regarding deaths when varying the CFP, $\phi$. A larger value of $\phi$ corresponds to a larger difference in the *M*(*t*) curves. In Fig. 4c, the effect of the individual and government actions reduces the height of the curve for the extreme CFP by four-fifths, while the reduction in plot (d) is less than 10in Fig. 5c, the reduction in the number of deaths is approximately one-fifth compared with that of the high CFP but is barely noticeable for the low CFP. This result highlights the importance of different measures (individual or collective) for reducing the effect of disease impacts.

For fitting the parameters, in Figs. 6 and 7, as mentioned in subsection entitled *Fitting using the health data from Chinese provinces*, some parameters are of good quality and promote modest to high confidence, while others are of low quality. However, we can highlight that our model is generally able to reproduce the disease outbreak rebound observed in the data, which was pointed out in our previous discussion. Additionally, it is worth noting that the free parameter $\beta _{01}$ is mainly fitted in the range from 0.5944 to 1.68, which is similar to the values reported by Lin et al.^8^.

The large differences in the values of $\sigma$ obtained in our data fittings probably indicate a contribution of the rapid onset of contagion once an individual is exposed, although they might also be due to differences in the population densities among the provinces of China. The contagion rate $\beta$ represents the likelihood that a susceptible individual (*S*(*t*)) is exposed (*E*(*t*)), and this depends on the outbreak intensity. However, once an individual is exposed, the time until they are infected is given by $\sigma$. This value contributes to a higher velocity of disease spread once an individual is exposed, thus indicating the importance of personal security measures.

The values of the parameters that determine the dynamics of disease spread are closely related to the period of time considered in the data, as well as the appearance of new variants. Viruses, such as SARS-CoV-2, mutate continuously and may have varying effects over time^15^. Thus, models need to adjust the values of the parameters accordingly and mainly consider the appearance of variants of concern (VOCs). Future work could consider a parameter factor that includes the variation rate of viruses or the appearance of VOCs, or they could even include more equations to represent several virus variants^12^.

Finally, for more detailed insights into the case of Hubei, we obtain the simulation results depicted in Fig. 8. The simulation time starts on December 1, 2019, using the timeline shown in Fig. 3 and the data from Hubei: the initial susceptible population $N_0= 1.14\times 10^{6}$, $\beta _{01} = \beta _{02} = 0.5$, as the value we obtain from the data fitting; the rest of the parameters are the same as in Table 1, and they are based on both government action and individual reaction to the perception of risk. The plots show the infected population *I*(*t*) in plot (a), the number of deaths *M*(*t*) in plot (b), the number of recovered individuals *R*(*t*) in plot (c) and the total number of cases in plot (d). Although we fit the values of the free parameters using a time series starting on January 22, the simulation qualitatively shows good agreement with the Hubei data. The peak of the number of contagious individuals occurs around day 50, which is close to the end of January, consistent with the results of Lin et al.^8^, who noted that there is a delay in values after processing the tests. Thus, the simulation results must be offset by approximately 15 days with respect to the data time series. However, the simulation shows an underestimation of the peak. The number of deaths *M*(*t*) is very similar to that reported in the data. With respect to the number of recovered individuals and total cases, the results show qualitatively good agreement but are quantitatively overestimated.

## Conclusions

In this paper, we present an SEIR model for computationally simulating the COVID-19 outbreak, and it considers the combined effect of governmental actions, public perception of contagion risk and the case fatality proportion (CFP). The outcome is a theoretical model that qualitatively reproduces the behavior expected for the COVID-19 outbreak based on the three scenarios of no governmental action or individual reaction due the perception of contagion risk, which is labeled as the naive category; only the individual reaction based on the perception of risk; and the combination of individual reaction and governmental actions. In all scenarios, we also consider low ($0.5\%$), middle ($1\%$), high ($2\%$), and extreme ($20\%$) values of CPF. The results of the simulations show that the influence of CFP variations is more important when considering the individual reaction in terms of the perception of risk. In one case, a disease outbreak rebound appears when only the individual reaction to risk is considered; however, this rebound effect disappears when government action is considered as a complementary measure. We conclude that individual actions with regard to the perception of contagion risk are more effective in reducing disease spread; however, government measures reinforce the reduction in spread when people relax after a reduction in the death rate. We deploy a maximum-likelihood method to fit health data from the 33 provinces of China provided by the Johns Hopkins University COVID-19 database. We fit the data for the free parameters of the initial population $N_0$, the inverse of the main latent period $\sigma$, and the baseline transmission rate $\beta _0$, which is split into two parameters, $\beta _{01}$ and $\beta _{02}$, acting at different times. The data fitting is reported for all 33 provinces. By adjusting certain parameters, the model can capture the transmission dynamics of the COVID-19 outbreak.
